# The most effective systemic treatment in dogs with sarcoptic mange: a critically appraised topic

**DOI:** 10.1186/s12917-023-03759-1

**Published:** 2023-10-05

**Authors:** Mirabela O. Dumitrache, Marie-Christine Cadiergues

**Affiliations:** 1 Department of Parasitology and Parasitic Diseases, Faculty of Veterinary Medicine, University of Agricultural Science and Veterinary Medicine Cluj-Napoca, Cluj-Napoca, Romania; 2 Department of Clinical Sciences, Université de Toulouse, ENVT, 31076 Toulouse, France; 3 INFINITy, Université de Toulouse, Inserm, CNRS, UPS, 31059 Toulouse, France

**Keywords:** Sarcoptic mange, *Sarcoptes scabiei* var. *canis*, Dog, Systemic treatment, Treatment efficacy

## Abstract

**Background:**

Sarcoptic mange is a common, pruritic parasitic skin disease of dogs. Due to its highly contagious character, it represents a potential veterinary and public health risk. Because of clinical similarity with other diseases, cross-antigenicity, and low sensitivity of available diagnostic methods, therapeutical trial is frequently used to confirm the disease. Considering the variety of available acaricidal molecules as well as the need to use the most effective treatment, the present paper reviews evidence comparing different types of systemic treatment of canine scabies.

**Results:**

Analysis of the results showed that afoxolaner, fluralaner and sarolaner as well as several macrocyclic lactones such as selamectin, moxidectin and milbemycin oxime can lead to parasitological and clinical cure.

**Conclusion:**

The similarity in the clinical and parasitological efficacy of these substances enhances the need for comparative studies, which could allow the identification of the most efficacious product.

## Background

Canine sarcoptic mange is a highly pruritic, non-seasonal, contagious and potentially zoonotic parasitic skin disease caused by the mite *Sarcoptes scabiei* var. *canis*. Dogs worldwide can be affected and no breed, age or sex predisposition has been demonstrated so far [1]. Contamination occurs through direct contact with an infested host or with a fomite, due to the capacity of the mite to survive and remain infective in the environment for up to 21 days [2, 3]. Clinical presentation is characterized by intense pruritus, alopecia, papules, erythema, scaling and crusting. The disease is often complicated by secondary pyoderma, which determines the extent of the lesions and contributes to the diversity of clinical presentation, ultimately complicating the diagnosis [2, 4]. Hypothetical diagnoses are based on the clinical presentation [1], while confirmation is obtained by demonstrating the presence of the parasite, its eggs or fecal pellets in skin scrapings [2] or positive serology [3] or a favorable response to a specific therapy [2, 5]. The diagnosis of sarcoptic mange may be complicated by several factors: the mites are difficult to find by skin scraping in early infections and in animals harboring low levels of parasitism, and therefore only 20 – 50% of skin scrapings from infested dogs are positive [1]. Serological diagnosis also has certain limitations : seroconversion occurs 3-5 weeks post infection or 1 to 3 weeks after the onset of the clinical signs, leading to false negative results if the animals are sampled too soon [2]. Furthermore, cross-antigenicity between *Sarcoptes scabiei*, house dust mites and storage mites has also been documented [2, 6]. Hence, therapeutical trial is one of the most frequently used methods to confirm the diagnosis in cases with suggestive clinical presentation but negative test results. In this context, only the most effective molecules should be used to confirm the diagnosis and/or to treat the disease. While the efficacy of contact topical acaricidal has always been considered to be limited and influenced by several factors, systemically acting acaricides, orally or topically administered, are widely recognized as efficient molecules and consequently preferred for treatment of the disease [3]. Macrocyclic lactones include several licensed molecules that have traditionally been used for the treatment of sarcoptic mange. More recently, other therapeutic options in the isoxazoline class have demonstrated their efficacy against *S. scabiei*. This paper critically reviews the literature on systemic treatments of canine scabies and uses available evidence to determine the most effective therapy, according to the relevant literature identified. The selected format is a critically appraised topic (CAT).

### Clinical scenario

The patient is a four-year-old German wirehaired pointer presented for a chronic, generalized, highly intense pruritus. The dog is used for hunting and is in close contact with two other dogs, who had started to develop similar clinical signs. A dermatological examination revealed the presence of alopecic and erythematous plaques, with scales and crusts distributed mainly on the ear pinnae, hocks and elbows. Microscopical examination of multiple skin scrapes revealed the presence of *S. scabiei* mites, enabling confirmation of sarcoptic mange. Given the range of licensed therapeutic options, the question is, which systemic molecule provides the most rapid clinical and parasitological cure.

### Clinical question

A population, intervention, comparison, outcome (PICO) question was formulated: “In dogs with sarcoptic mange, which systemic treatment is the most efficacious?”

P (population) = dog with sarcoptic mange.

I (intervention) = systemic treatment.

C (comparison) = different systemic molecules.

O (outcome) = clinical and parasitic resolution.

Preferred study type = clinical trials.

### Search strategy

A literature review was conducted to identify different types of treatments using the following criteria: ((dogs OR canine) AND (scabies OR Sarcoptes scabiei OR sarcoptic mange) AND (treatment)). Two electronic databases were searched (PubMed and CAB), with no time limit up to 4^th^ October 2022. In this study we followed the Preferred Reporting Items for Systematic Reviews and Meta-Analysis (PRISMA) updated guideline for systematic review stated in 2020 [7].

### Quantity and quality of evidence

**Fig. 1:**
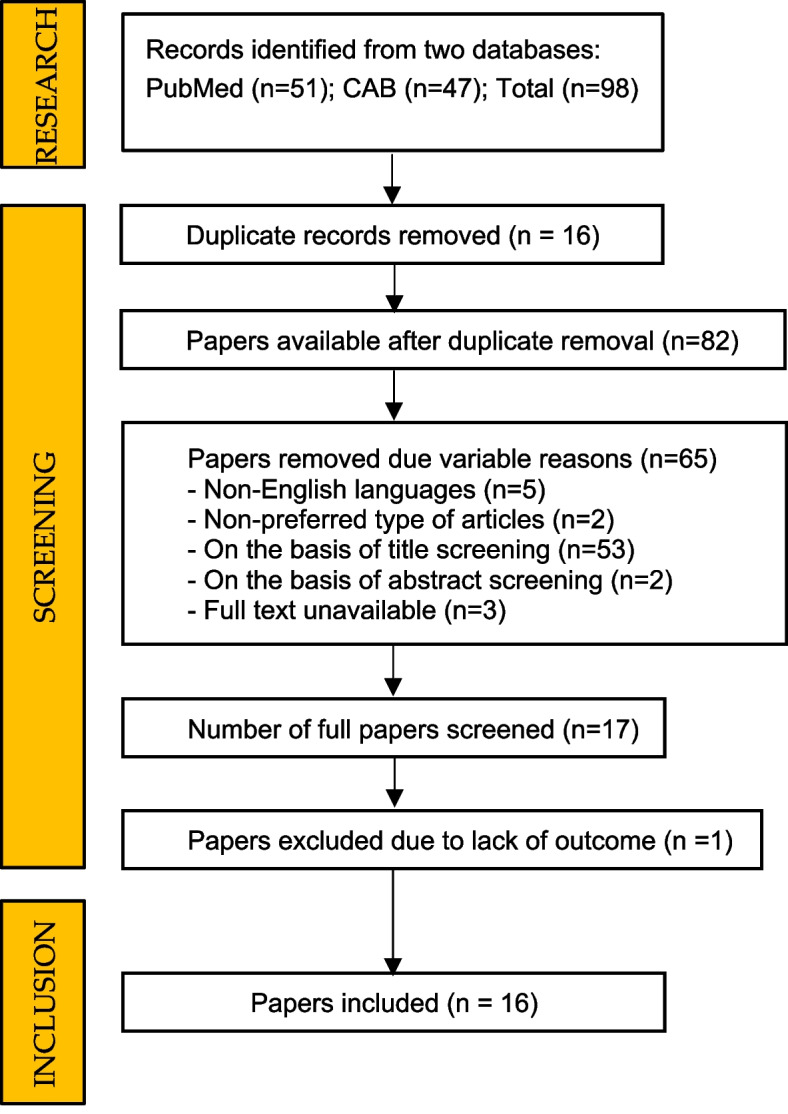
Search strategy and selection process flow-chart

The search produced 51 results in PubMed and 47 in CAB. The preferred language was English. Among the full-text potentially eligible articles, only 16 met the eligibility criteria, including relevance to our PICO question and multi-animal inclusion, and were included in our review, whereas case reports and case series articles were excluded (Fig. 1).

The data that highlight the quality of the evidence, such as the journals in which the articles were published, the number of enrolled subjects, and the type of study are summarized in Table 1. All the papers included in this study were published in international peer-reviewed journals. The quality of evidence presented in the selected papers was very uneven. The main factors responsible were variable numbers of the enrolled subjects, as well as the study design. However, in all cases, the quality was considered sufficient to contribute to the conclusion of the PICO.

**Table 1:** Summary of the quality of evidence

**Journal/Publication year**	**Type of study**	**Number of subjects**	**Reference**
American Journal of Veterinary Research/1984	Prospective with control group, single center	298	[8]
The Canadian Veterinary Journal/1996	Prospective with no control group, multi-center	27 and 14 respectively	[9]
The Canadian Veterinary Journal/1997	Prospective with no control group, single center	120	[10]
Veterinary Parasitology/2000	Prospective, blind, randomized, with control group, multi-center	42	[11]
Veterinary Parasitology/2000	Prospective, blind, randomized, with control group, multi-center	207	[12]
Veterinary Parasitology/2000	Prospective no control group, single center	41	[13]
Parasitology Research/2005	Prospective, blind, randomized, with control group, multi-center	53	[14]
Australian Veterinary Journal/2006	Prospective, blind, randomized, with control group, single center	30	[15]
Veterinary Research Communications/2011	Prospective with control group, single center	22	[16]
Veterinary Parasitology/2016	(1) Laboratory study, blind, randomized, placebo, with control group, single center (2) Prospective, blind, randomized with control group, multi-center	(1) 44 and (2) 79 respectively	[17]
Parasite/2016	Prospective, randomized, with control group, single center	20	[18]
Veterinary Dermatology/2016	Prospective, no control group, single center	17	[4]
Parasites & Vectors/2016	Prospective, double-blind, randomized, with control group, single center	29	[19]
Parasite/2018	Prospective, double-blind, randomized, no control group, multi-center	80	[20]
Parasites & Vectors/2020	Prospective, single-blind, with control group, multi-center	135	[3]
The Canadian Journal of Veterinary Research/2020	Prospective, with control group, single center	142	[21]

## Results and discussion

Out of the 16 studies included in our review, nine tested products that only contained macrocyclic lactones as miticide [8–16], isoxazoline alone was tested in five studies [3, 4, 17–19], the efficacy of isoxazoline compared with an association of an isoxazoline plus a milbemycin was analyzed in two studies [20, 21], and isoxazolines and a milbemycin were compared in one study [17]. The majority of studies used positive controls [3, 11, 13–16, 19, 20], but four of the studies had no control group [4, 9, 10, 13], and in five of the studies, treated groups were compared with a placebo [8, 11, 17, 19] or with untreated groups [18].

Positive skin scraping was the *sine qua non* inclusion criterion in all but five studies described in four articles [8–10, 13] and was also the technique used to evaluate the efficacy of the parasitological cure. Animals enrolled and still present at the end of the study were declared parasitologically cured in 12 of the studies [3, 4, 8, 11, 12, 14–20]. Interestingly, one study demonstrated that adding vitamin E and selenium to a protocol of a weekly subcutaneous administration of ivermectin improved efficacy from 82% to 100% by day 28 [16]. Although the use of ivermectin may be preferred due to its low cost, the high probability of side effects in some breeds, the fact the product is not licensed in some countries, as well as the reported refractory cases should be taken into account [22].

The majority of substances tested led to a marked improvement in skin lesions and pruritus. Dogs were declared clinically cured in five of the studies, three of which tested macrocyclic lactones. In the first of these studies, 2 mg/kg of milbemycin oxime was administrated orally two or three times, either weekly or at two week intervals [9]. However, the timing of the clinical evaluation relative to the time treatment was administered was not mentioned in the study, and what is more, a relapse of sarcoptic mange was reported in two dogs in the same study [9]. In the second of the five studies, a topical 0.5% ivermectin solution was applied on day 1 and day 15 of the study, at a dose of 500 μg/kg. The dogs were evaluated as clinically cured by day 150 [10]. The third study evaluated the efficacy of oral and/or subcutaneous weekly administration of moxidectin until clinical remission (3-6 weeks). Thirty-seven of the enrolled animals were evaluated as clinically cured within this 3-to-6-week time frame. Among the original total of 41 dogs, seven exhibited side effects, and in three animals, treatment was discontinued for this reason [13]. The other two studies that reported a clinical cure tested molecules belonging to the more recently discovered isoxazoline class. The animals in the study using oral or topical fluralaner and oral sarolaner were considered clinically cured by day 84 [3] while animals treated with afoxolaner, and afoxolaner plus milbemycin oxime were declared cured by day 56 [21]. A slightly more rapid disappearance of clinical symptoms was reported with the medication in which milbemycin oxime was added to afoxolaner, despite the fact that the dose of milbemycin oxime added in the tested product was below the therapeutic dose recommended for sarcoptic mange [20]. These results are consistent with those obtained previously in a similar study [20], except that in the study that tested oral administration of milbemycin oxime, the dogs were followed up for longer, i.e. between 42 and 150 days. It has already been reported that resolution of alopecia in dogs with sarcoptic mange increases significantly after day 56, and that a period of four weeks between administration of the treatment and clinical evaluation is most likely too short to allow complete resolution of the lesions [20]. A longer observation period would therefore improve the reliability of clinical cure.

Five studies tested the efficacy of selamectin and/or imidacloprid plus moxidectin, and showed that two monthly applications of either of the two spot-on products can lead to parasitological and clinical cure. However, results differed regarding the time of the appearance of the parasitological cure, which varied between day 22 and day 60 [11, 12, 14, 15]. Moreover, the only study of the efficacy of a macrocyclic lactone against an isoxazoline showed that sarolaner was slightly more effective, reaching 100% efficacy by day 60, compared to 96% efficacy of the product containing imidacloprid plus moxidectin [17].

A single oral dose of fluralaner administered 10 to 20 minutes after food, or topically, was shown to lead to parasitological cure and to a significant improvement in skin lesions and pruritus by day 28 [4, 19]. All the other tests of isoxazolines, sarolaner, afoxolaner, and afoxolaner plus milbemycin oxime, demonstrated high efficacy against canine sarcoptic mange after a monthly dose, and all the products tested led to parasitological cure [16, 17, 19, 20].

It is known that successful treatment of canine sarcoptic mange depends to a great extent on the product’s ability to immediately eliminate all active mites. The effect must last long enough to prevent renewed contamination from eggs laid by hatched larvae, fomites or through contact with other infected animals [3]. Interestingly, none of the studies we reviewed reported treatment of the environment. However, when required (multiple animals involved, with no possibility of housing them individually), in-contact dogs were also treated.

The main results of all the studies are summarized in Table 2.

**Table 2:** Summary of the key results

**Tested medication**	**Patients**	**Study design**	**Outcome and key results**	**Study weakness**	**Reference**
Ivermectin s.c. vs. placebo	298 mixed-breed dogs, housed in 12 pens containing 10 to 60 dogs; 70% severe form; 25% moderate form; 5% mild form.	Diagnostic method: skin scrapes and/or clinical. Ivermectin, 200 μg/kg: Group A (*n*=20), Group D (*n*=256): D0; D14 Group B (*n*=12), Group C (*n*=10): D14. Vehicle/Sterile solution 0.9% NaCl: Group B (*n*=12), Group C (*n*=10): D0.	% efficacy D14: Group A=95%; Group B=42%; Group C=30%. Group A, B, C: parasitological cure D14 following the 2^nd^ ivermectin treatment. Group D: D42 clinically similar to Group A. Major improvement in skin lesions and pruritus in all groups.	Animals enrolled only based on clinically compatible signs. Out of the total number of enrolled animals (*n*=298), only a medium number of animals (*n*=42) were monitored systematically. D42 data refers to 5 dogs in group A and B and 3 dogs in group C.	[8]
Oral milbemycin oxime	Phase one: 27 owned dogs, 3 mongrels and 24 pure breeds.	Diagnostic method: skin scrapes (*n*=22) and/or clinical (*n*=5). Milbemycin oxime, 2 mg/kg: D0, D7, D14. All in-contact dogs were treated.	50% to 75% reduction in pruritus after the 3rd dose. Two dogs relapsed after 2.5 months. D28: clinical cure.	Uncontrolled study Animals enrolled only based on clinical compatible signs. Medium or small number of animals. No control test for parasites during and/or at the end of the study. Lack of quantitative outcome.	[9]
	Phase two: 14 dogs, Beagle, housed in the same facility in groups of 4.	Diagnostic method: skin scrapes (performed randomly) and/or clinical. D0, D14, milbemycin oxime, 2 mg/kg.	All dogs were clinically normal at the end of the study and 10 months after. One dog died due to events unrelated to the therapy.		
Topical ivermectin 0.5%	120 dogs, 118 mixed-breed, housed into 35 pens containing from 2 to 5 dogs/pen; severe in 15 dogs; moderate in 70 dogs; mild in 35 dogs.	Diagnostic method: skin scrapes (performed randomly) and/or clinical and/or fecal flotations (performed randomly) 0.5% pour-on ivermectin, 500 μg/kg: D1 and D15 Ivermectin, s.c., 300 μg/kg on D1 and D15 to all animals entering or departing the shelter	90 of the 120 dogs present on D1 were still at the shelter on D150. D15: Major improvement in skin lesions and pruritus in all dogs. D30: clinical remission observed in all but 2 dogs. D150: clinical remission in all dogs (*n*=90)	Uncontrolled study Animals enrolled only based on clinically compatible signs. Control tests were conducted randomly and irregularly. Lack of quantitative outcome.	[10]
Topical spot-on selamectin 12% vs. placebo	30 dogs, 17 mixed-breed and 13 purebreds from kennels (USA), 12 purebred dogs from hunting kennels (Italy). Dogs were housed in purpose-designed buildings in a single site in each country.	Diagnostic method: skin scrapes. Selamectin, min. 6 mg/kg or vehicle: D0 and D30 Dogs were randomly allocated to vehicle (15 dogs in USA; 6 in Italy) or selamectin (15 dogs in USA; 6 in Italy) group.	3 dogs were withdrawn from the study (2 from vehicle group Italy and 1 from selamectin group USA). Parasitological cure in selamectin group: D29/30, Italy and D44, USA. The mean count mite in the selamectin treatment was significantly lower than that in the vehicle treatment in both studies on all evaluation days. Major improvement in skin lesions and pruritus in both selamectin groups.	Medium or small number of animals.	[11]
Topical spot-on 12% selamectin vs. N-(mercaptomethyl) phthalimide S-(0,0-dimethyl phosphorodithioate), dip solution, in USA; vs. phosmet, sponge-on, in UK; vs. amitraz, body-wash solution, in Italy.	207 dogs, purebred and mix-breed, housed both indoors and outdoors, in USA and Europe (UK and Italy)	Diagnostic method: skin scrapes. Selamectin, min. 6 mg/kg^:^ D0 and D30; N-(mercaptomethyl) phthalimide S-(0,0-dimethyl phosphorodithioate), 7.8 mL l-1, 1 to 8 doses, D0, D14, D21, D28, D35, D42, D49, D56; phosmet 0.09%, 1 to 4 doses, every 14 days; amitraz 0.025%, 2 to 4 doses, D0, D7, D30, D37. Animal allocation: USA: Selamectin *n*=54; positive control (PC) =19. Europe: Selamectin *n*=68; positive control (PC) =37.	29 dogs did not complete the study. The efficacy of selamectin was >95% by day 30, and 100% by day 60. Improvement in skin lesions and pruritus in all treatment groups.	The reason for the withdrawal from the study was not given for all the animals.	[12]
Oral or s.c. moxidectin.	41 owned dogs of various breeds	Diagnostic method: skin scrapes (*n*=3) or serological (*n*=17) or therapeutical (*n*=21). 0.2–0.25 mg/kg moxidectin, administrated once / week until clinical signs were gone (3-6 weeks): orally (*n*=4) or subcutaneously (*n*=31), the first dose was given subcutaneously and the following orally (*n*=6). All in-contact dogs were treated.	4 dogs were withdrawn from the study (3 because of side effects). 7 dogs treated s.c. showed side effects. Clinical cure in all remaining 37 dogs.	Uncontrolled study. Lack of quantitative outcome. Small or medium number of animals.	[13]
Topical spot on: imidacloprid 10% + moxidectin 2.5% vs. 12% selamectin.	58 owned dogs	Diagnostic method: skin scrapes and clinical criteria. Imidacloprid 10% + moxidectin 2.5% (*n*=27) and 12% selamectin (*n*=26): D0 and D28.	Five dogs were withdrawn due to events unrelated to treatment. Parasitological cure rate on D56 was 100% in both study groups and >96% of the dogs in both groups were clinically cured or improved. One dog treated with selamectin showed side effects.	Medium number of animals.	[14]
Topical spot on: imidacloprid 10% + moxidectin 2.5% vs. 12% selamectin.	30 owned mixed-breed dogs, housed individually	Diagnostic method: skin scrapes. Imidacloprid 10% + moxidectin 2.5% (*n*=15) and 12% selamectin (*n*=15): D0 and D28.	One dog treated with Imidacloprid 10% + moxidectin 2.5% was withdrawn due to events unrelated to treatment. Parasitological cure starting from D22 in both groups. Marked improvement in skin lesions and pruritus in both treatment groups by D36.	Three dogs in the selamectin group had concurrent demodicosis. Medium number of animals.	[15]
Ivermectin s.c. vs. ivermectin s.c. + vitamin E + selenium.	31 dogs (9 healthy and 22 dogs infested with sarcoptic mites)	Diagnostic method: skin scrapes. Collection of blood samples to estimate oxidative stress indices, hematology, and biochemical panels: D0 and D28. 1% ivermectin s.c. 0.2 mg/kg (*n*=11, Group 2), 1% ivermectin s.c. 0.2 mg/kg + (tocopherol 50 mg + Se 1.5 mg/mL) 0.5 mL/20 kg i.m. (*n*=11, Group 3): weekly, three times. Antihistamines: 5-7 days for all infested dogs.	Parasitological cure rate D28: Group 2 = 82%; Group 3 = 100%. Marked improvement in skin lesions in both treatment groups by D28, with maximum recovery in Group 3. D28: significantly alleviated malonyldialdehyde in Group 2 and 3; CAT and SOD were significantly elevated in both groups; GSH was significantly elevated in group 3.	Small number of animals. No data provided on the animals’ housing.	[16]
Oral sarolaner vs. placebo.	Study 1: 44 owned mixed breed dogs, individually housed.	Diagnostic method: skin scrapes. Sarolaner, 2 mg/kg (*n*=22), Placebo (*n*=22): D0 and D30. I.m. or s.c. dexamethasone (0.4 mg/kg, 3 adm. /week for two weeks) in 10 dogs from each group, before or after the first treatment.	2 dogs in the sarolaner group and 4 in the placebo group were withdrawn due to events unrelated to treatment. The efficacy in the sarolaner group compared to the placebo group or relative to the pre-treatment mean was >99% in all post treatment evaluations. Parasitological cure for sarolaner group vs. placebo group on D30: 100% vs 65%.	Immunosuppression therapy during the study.	[17]
Oral sarolaner vs spot-on imidacloprid + moxidectin.	Study 2: 79 primary and 45 supplementary (in-contact) owned dogs.	Diagnostic method: skin scrapes. Sarolaner, 2mg/kg (*n*=53), imidacloprid/moxidectin group (*n*=26): D0 and D30. All in-contact (supplementary) dogs were treated with the same molecule and frequency as the primary dog: Sarolaner, 2 mg/kg (*n*=26), imidacloprid/moxidectin group (*n*=19): D0 and D30.	Parasitological cure in the sarolaner group vs. imidacloprid + moxidectin group, on D60: 100% vs. 96%. Marked improvement in skin lesions and pruritus in both treatment groups	One dog in the imidacloprid/moxidectin group was under-dosed at D30. 7 dogs in the sarolaner group received concomitant antimicrobial and/or anti-inflammatory treatment, due to various medical events.	
Oral afoxolaner.	20 owned mixed-breed dogs.	Diagnostic method: skin scrapes. Afoxolaner, min. 2.5 mg/kg (*n*=11): D0 and D28. Control group (*n*=11): not treated.	Parasitological cure, by day 28: afoxolaner group, efficacy 100%, Control group 10%. Significant improvement in skin lesions and pruritus in the afoxolaner group by day 56.	Small number of animals.	[18]
Oral fluralaner.	17 owned dogs.	Diagnostic method: skin scrapes. Fluralaner, min. 25 mg/kg: D0.	Parasitological cure by day 14. Significant reduction in lesions by day 14. Significant reduction in pruritus by days 14 to 21.	Uncontrolled study Medium number of animals.	[4]
Oral and topical spot-on fluralaner vs. placebo.	29 owned mixed-breed dogs.	Diagnostic method: skin scrapes. Oral fluralaner min. 25 mg/kg (*n*=9), topical fluralaner 25 mg/kg (*n*=11), topical saline solution (*n*=9): D0. All the dogs from the same household were enrolled in the same treatment group.	3 dogs in the topical fluralaner group did not complete the study due to events unrelated to treatment. Parasitological cure by D28: 100% in treated groups, 33.3% in placebo group. Improvement in skin lesions and pruritus in both treated groups by D28.	Small number of animals. No post-treatment clinical evaluation of the control group.	[19]
Oral afoxolaner and oral afoxolaner + milbemycin oxime.	80 owned, mixed-breed and purebred dogs.	Diagnostic method: skin scrapes. Oral afoxolaner (*n*=38) and oral afoxolaner + milbemycin oxime (*n*=27), 2 administrations: D0 and D26-D30.	14 dogs were excluded due to non-compliance and 1 was erroneously enrolled. Parasitological cure by two months after the first treatment: 99.7% afoxolaner group, 100% afoxolaner + milbemycin oxime group. Significant improvement in pruritus and skin lesions two months after the first treatment	Uncontrolled study No data provided regarding the group assignment of excluded animals.	[20]
Oral and topical spot-on fluralaner and oral sarolaner.	Owned, mixed-breed and purebred dogs: 135 primary and 19 supplementary (in-contact) dogs.	Diagnostic method: skin scrapes. Oral and topical spot-on fluralaner, min. 25 mg/kg: D0, and oral sarolaner, min. 2 mg/kg: D0 and D28. All the dogs from the same household were enrolled in the same treatment group.	9 dogs were excluded due to non-compliance (3 dogs in the topical fluralaner group, 1 dog in the oral fluralaner group, and 5 dogs in the oral sarolaner group). Parasitological cure, efficacy 100% by D56 in all groups. Substantial improvement in pruritus and skin lesions in all groups by D56. Clinical and parasitological cure in all groups by D84.	2 dogs, 1 in the sarolaner group, and 1 in the fluralaner spot-on group, received oral antibiotics.	[3]
Oral afoxolaner and oral afoxolaner + milbemycin oxime.	142 owned dogs.	Diagnostic method: skin scrapes. Oral afoxolaner + milbemycin oxime (*n*=96) and oral afoxolaner (*n*=46): D0.	Clinical cure in both groups by D56. Absence of pruritus in the afoxolaner + milbemycin oxime group by D28 and in the afoxolaner group by D56.	No data provided on parasitological cure. No data provided on group allocation. Not mentioned if it is a blind study.	[21]

*Abbreviations: vs* versus, *s*.*c*. sub-cutaneous, *i*.*m*. intramuscular, *D* day, *mg* milligram, *kg* kilogram, *mL* milliliter, *min*. minimum

## Conclusion and implication for practitioners

Our analysis of scientific evidence allowed us to conclude that two administrations one-month apart of topical spot-on 10% imidacloprid plus 2.5% moxidectin or 12% selamectin as well as oral sarolaner, afoxolaner, and afoxolaner plus milbemycin oxime, or one administration of topical or oral fluralaner, can lead to parasitological cure plus at least significant improvement of clinical lesions and pruritus. Moreover, no side effects were reported. The oral treatment with fluralaner allowed the most rapid parasitological cure, by day 14 all animals having negative skin scrapings. It is also the substance that was the fastest in resolving clinical signs within 21 days after a single dose. However, this data should be interpreted with caution, taking into account that weekly control during one month after the administration of the treatment were not performed in all studies.

Due to the similar clinical and parasitological efficacy of these substances, comparative studies using a similar design are now needed to identify the most efficient product. Previous studies have been demonstrated that clinical healing and absence of parasites at skin scrapings occur weeks to months before obtaining negative serum *Sarcoptes*-IgG tests [5]. However, adding to the classical evaluation methods a long-term serological surveillance might add new useful data and provide for clinicians a complete data set for tested molecules.

Thus, when deciding which product to use, other criteria may need to be taken into consideration such the safety of macrocyclic lactones in some breeds, the fact the extent and severity of skin lesions may interfere with the application of the topical products, the availability of licensed products, the frequency of administration, the compliance of the patient and the agreement of the owner regarding the method of administration.

## Data Availability

All data analysed during this study are included in this published article.
